# Non-contact tonometry: predicting intraocular pressure using a material—corneal thickness—independent methodology

**DOI:** 10.3389/fbioe.2024.1406870

**Published:** 2024-07-25

**Authors:** Elena Redaelli, Begoña Calvo, Jose Felix Rodriguez Matas, Giulia Luraghi, Jorge Grasa

**Affiliations:** ^1^ Aragón Institute of Engineering Research (I3A), Universidad de Zaragoza, Zaragoza, Spain; ^2^ Centro de Investigación Biomecánica en Red en Bioingeniería, Biomateriales y Nanomedicina (CIBER-BBN), Zaragoza, Spain; ^3^ LaBS, Department of Chemistry, Materials and Chemical Engineering “Giulio Natta”, Politecnico di Milano, Milan, Italy

**Keywords:** intraocular pressure, fluid structure interaction simulation, central corneal thickness, corneal mechanical properties, energetic balance, Corvis ST

## Abstract

**Introduction:** Glaucoma, a leading cause of blindness worldwide, is primarily caused by elevated intraocular pressure (IOP). Accurate and reliable IOP measurements are the key to diagnose the pathology in time and to provide for effective treatment strategies. The currently available methods for measuring IOP include contact and non contact tonometers (NCT), which estimate IOP based on the corneal deformation caused by an external load, that in the case of NCT is an air pulse. The deformation of the cornea during the tonometry is the result of the coupling between the IOP, the mechanical properties of the corneal tissue, the corneal thickness, and the external force applied. Therefore, there is the need to decouple the four contributions to estimate the IOP more reliably.

**Methods:** This paper aims to propose a new methodology to estimate the IOP based on the analysis of the mechanical work performed by the air jet and by the IOP during the NCT test. A numerical eye model is presented, initially deformed by the action of a falling mass to study the energy balance. Subsequently, Fluid-Structure Interaction (FSI) simulations are conducted to simulate the action of Corvis ST.

**Results and discussion:** The new IOP estimation procedure is proposed based on the results of the simulations. The methodology is centred on the analysis of the time of maximum apex velocity rather than the point of first applanation leading to a new IOP estimation not influenced by the geometrical and mechanical corneal factors.

## 1 Introduction

One of the objectives of the human eye is the convergence of incoming light rays from the external environment onto the retina, situated on the posterior segment of the eye. The retina establishes a connection with the brain through the optic nerve, which transmits visual signals. The aqueous humor, a clear fluid that fills the ocular chamber, maintains the eye’s structural integrity and is pressurized within a range of 8–20 mmHg in healthy conditions, referred to as intraocular pressure (IOP) (Wang et al., 2018). The equilibrium between the production and drainage of aqueous humor governs the IOP, which, when elevated, poses a substantial risk factor for the development of glaucoma, a collective term for a group of eye disorders that can lead to irreversible vision loss (Jordanova et al., 2022). The vision impairment associated with glaucoma is primarily attributed to the degeneration of the optic nerve, which is a consequence of elevated IOP. Glaucoma is a leading cause of blindness worldwide, after cataracts in underdeveloped countries and after senile degeneration of the macula in developed countries (Jordanova et al., 2022). In 2020, the disease affected about 80 million people globally, and this figure is projected to rise to 111 million by 2040 (Brusini et al., 2022). Because glaucoma has a gradual onset and lacks noticeable symptoms until late stages, it is often referred to as a “silent disease” (Brusini et al., 2022). In this context, reducing IOP is the primary option available for managing the pathology and preventing blindness (Sharma et al., 2020; Eliasy et al., 2022).

Accurate and repeatable IOP measurements are the key to diagnose the pathology in time and to provide for effective treatment strategies. However, currently available methods are indirect, they only provide an estimation of IOP (Brusini et al., 2021; Brusini et al., 2022; Silva and Lira, 2022), and the evaluation of their precision and accuracy is a critical field of research.

Ophthalmologists use a variety of techniques to measure IOP, including Goldmann applanation tonometry (GAT) (Goldmann and Schmidt, 1957) and Non Contact Tonometry (NCT) such as Corvis ST and Ocular Response Analyzer (ORA). The gold standard is GAT: it involves the use of a probe that is gently pressed against the cornea to flatten a small area, creating a uniform surface of known size. A calibrated force is then applied to the probe, and the amount of force required to flatten the cornea is measured. The estimation of IOP in GAT is based on the Imbert-Fick law (Imbert, 1885): it is a modification of the Maklakoff law (Maklakoff, 1885), and states that an external force against a sphere equals the pressure in the sphere multiplied by the area applanated by the external force. The validity of the law requires that the sphere should be perfectly spherical, dry, perfectly flexible and infinitely thin (Sharma et al., 2020), conditions that are not fulfilled in the cornea (Wu et al., 2020; Brusini et al., 2021). Other methods to measure the IOP are the ORA and the Corvis ST, which are NCTs. Their purpose is to obtain IOP measurements with minimal invasion of the eye (Silva and Lira, 2022); the idea is the same as GAT but the deformation of the cornea is obtained by a high-velocity air jet, without the need of contact. The difference between ORA and Corvis ST is that in the former, the air pressure at the outlet of the device varies among patients, while in the latter it is always the same; however, both of them provide an estimation of the IOP based on the first applanation time of the cornea during the air jet. In addition, Corvis ST is equipped with a Scheimpflug camera which gives 140 images of the central section of the cornea over the 30 ms of the air jet.

Although GAT and NCT are the most widely used instruments to estimate the IOP (Silva and Lira, 2022), their accuracy is significantly affected by the thickness of the cornea and the mechanical properties of corneal tissue (Ariza-Gracia et al., 2015; Asaoka et al., 2015; Greene et al., 2016). It has been proved (Ajazaj et al., 2018), for instance, that IOP measurements following LASIK refractive surgeries for the correction of myopia may be unreliable due to changes in central corneal thickness (CCT) and due to the applanation of the corneal surface after surgery. To mitigate the risk of obtaining falsely low IOP applanation readings after LASIK, adjustments should be made to the measured IOP, as highlighted in studies such as (Kohlhaas et al., 2006; Ajazaj et al., 2018; Helmy and Hashem, 2020). Other studies demonstrated that the biomechanical properties of the eye affect the measurement of IOP (Liu and Roberts, 2005; Kaushik et al., 2012; Aoki et al., 2023). The corneal deformation during tonometry is the result of the coupling between four factors: the IOP, the mechanical properties of the corneal tissue, the corneal geometry (in particular, its thickness), and the external force applied (Ariza-Gracia et al., 2015). The first applanation time used to estimate the IOP in NCT, could be different in patients with the same IOP because those patients can have different corneal mechanical properties or different corneal geometries.

Corvis ST provides a corneal-corrected IOP measurement, the bIOP, designed to exclude the influence of central corneal thickness and age (Joda et al., 2016; Eliasy et al., 2022). There is still a need to reduce its dependence on corneal mechanical properties. bIOP derives from an algorithm correlating various dynamic corneal response parameters obtained through structural numerical simulations of Corvis ST (Eliasy et al., 2022). However, the simulations assume a constant air pressure over the corneal apex which does not depend on corneal deformation like in the real scenario. Furthermore, the numerical model assumes the mechanical properties of the corneal tissue solely age-dependent. While age is a factor, individuals of the same age may exhibit different mechanical properties in their corneal tissue (Ariza-Gracia et al., 2015). As a consequence of this simplification, the algorithm proposed can be improved. A methodology to reliably estimate the IOP not influenced by the mechanical properties and geometry of the cornea is still missing.

This paper aims to propose a new methodology to estimate the IOP. The methodology is based on the analysis of the internal and external energies impacting the anterior corneal surface during the first 10 ms of the corneal deformation during the air puff.

The paper is organized as follows. In the materials and methods section, the numerical model of the eye is presented. Two different loading conditions are proposed: a simplified model of a mass falling under the gravity effect - which allows for a easier examination of the external energy exerted on the eye - and a Fluid-Structure Interaction (FSI) simulation simulating the air puff of Corvis ST. In the same section, the energetic analysis is outlined highlighting the region of interest (the anterior corneal surface) and the energies playing a role in the deformation of the eye. In the following section, the results of the energetic analysis are presented for both the loading cases. Then, the new methodology to estimate the IOP is proposed. The paper concludes with a discussion of the obtained results.

## 2 Materials and methods

### 2.1 Numerical model

**FIGURE 1:**
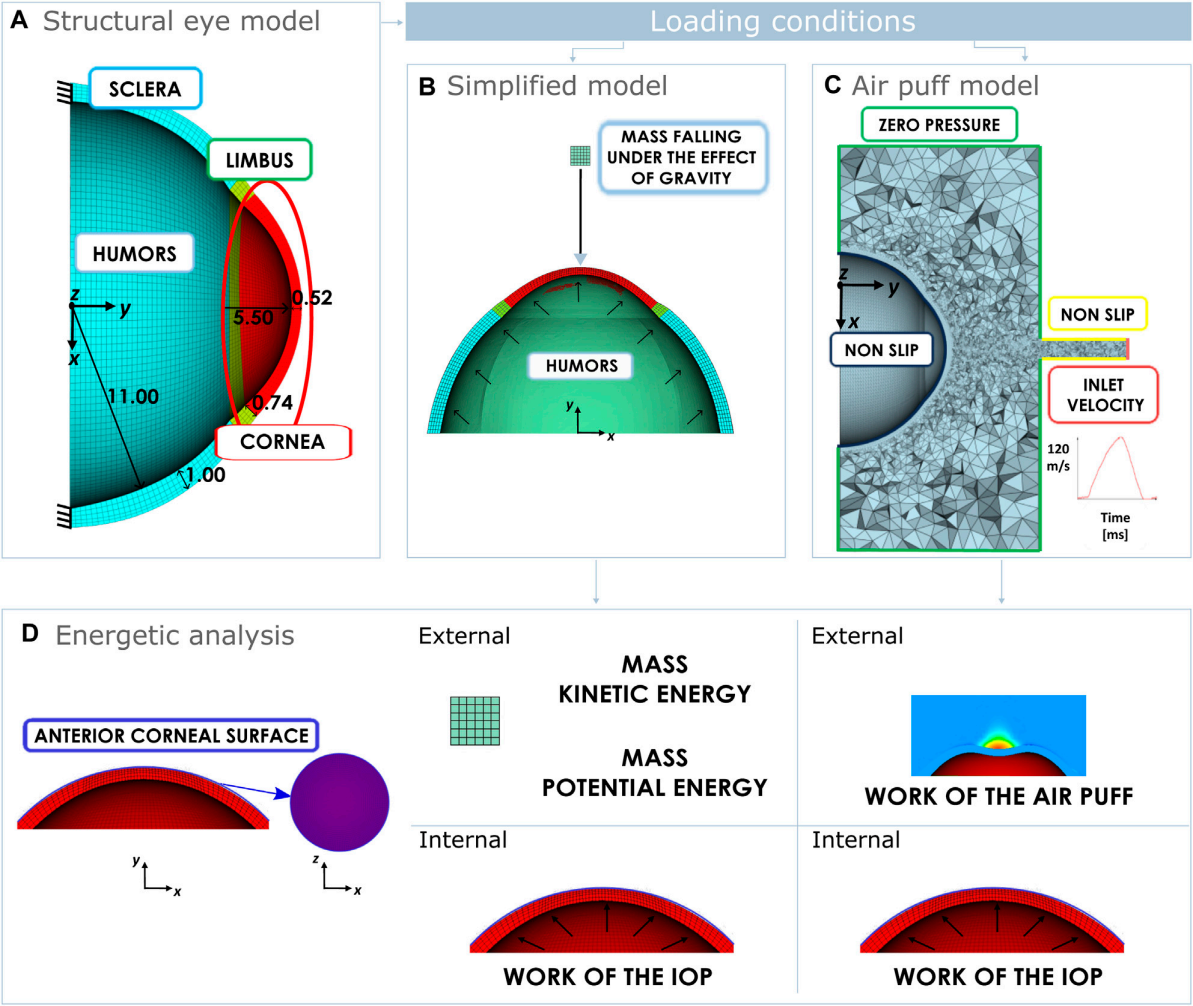
**(A)** Structural eye domain with some dimension in mm and boundary conditions. Loading conditions of the eye: **(B)** Mass falling under the gravity effect. **(C)** Fluid domain simulating the air puff and boundary conditions. **(D)** Energetic analysis performed on the anterior corneal surface in both cases. In case of a falling mass, the anterior corneal surface is loaded externally by the mass kinetic energy and the mass potential energy. In case of an air puff, the anterior corneal surface is loaded externally by the work of the air puff. Internally in both cases the cornea is loaded by the work of the IOP.

The structural model of the eye used in the simulations is depicted in Figure 1A. It comprises cornea, limbus, sclera and humors. The humors are modelled as an incompressible fluid pressurized at IOP. The cornea and the limbus are described as anisotropic, nearly incompressible, hyperelastic materials, to account for the influence given by the network of collagen fibres. The cornea is described with two families of mutually orthogonal collagen fibres assumed perfectly aligned with the nasal-temporal (NT) and superior-inferior (SI) direction and the limbus is described with one circumferential family of fibres. In both cases, the Holzapfel-Gasser-Ogden (Holzapfel et al., 2000) constitutive model is used. Eq. (1) shows the isochoric contribution of this model to the free energy.

$$\bar{\Psi }=C_{10}\left({\bar{I}}_{1}-3\right)+\frac{k_{1}}{2k_{2}}\underset{i=4,6}{\sum }e^{k_{2}{\left({\bar{I}}_{i}-1\right)}^{2}}$$ (1)

where $C_{10}$ [MPa] is a material parameter related to the extracellular matrix behaviour, $k_{1}$ [MPa] refers to the stiffness of the fibres and $k_{2}$ [-] models their non-linearity. $I_{1}$ is the first invariant of the right isochoric Cauchy-Green stress tensor, while $I_{4}$ and $I_{6}$ are respectively the fourth and the sixth pseudo-invariants related to the fibres stretch. The sclera is modelled as a nearly incompressible hyperelastic isotropic material with a Neo-Hookean formulation Eq. (2).

$$\bar{\Psi }=C_{10}\left({\bar{I}}_{1}-3\right)$$ (2)

More details on the geometry and materials functions used in the structural model can be found in our previous work (Redaelli et al., 2024). The geometry is meshed using software ANSA Pre Processor v23.01 (BETA CAE Systems, Switzerland) with hexahedral solid elements. The structural eye model presented corresponds to a pressurized configuration, therefore, in the first step of each simulation, the zero pressure configuration of the eye is computed following the iterative algorithm presented previously (Ariza-Gracia et al., 2016). Once the zero-pressure configuration is found, the incompressible fluid of the humors is pressurized to obtain the reference configuration of the eye.

To study the energetic balance of the eye during NCT, we preliminary investigated the behaviour of a mass falling under the effect of gravity as presented in Figure 1B. A mass of 2.02 g modelled as a rigid cube with a side length of 1.5 mm, falls onto the cornea from a height of 8 mm with respect to the apex of the cornea. This simplified model is useful to comprehend the energetic balance of the eye when subjected to an external load. In this case, the total mechanical energy of the mass transmitted to the cornea during the impact is easy to calculate (corresponding to the sum of potential and kinetic energy of the mass).

Once the simplified model was analyzed, 3D FSI simulations were run to reproduce *in silico* the NCT. Since during the NCT, an air puff hit a cornea causing its deformation, FSI simulations are the best numerical approach to model the phenomenon (Ariza-Gracia et al., 2018); where the structure (the eye) and the fluids (the air sorrounding the eye and the humors) are coupled. The fluid domain of the FSI, along with the boundary conditions are depicted in Figure 1C. The inlet velocity corresponds to the velocity of the air puff at the outlet of the nozzle of Corvis ST.

In both cases, after the pressurization, a deformation of the eye occurs caused by the action of an external agent. In the first phase of the impact, small deformations occurs, then after the corneal applanation, the cornea continues to deform through bending, resulting in larger deformations.

The simulations were implemented in the finite-element solver LS-Dyna R14.0 (ANSYS, 71 Inc., Canonsburg, PA, United States) (Dev, 2024) and performed using an Intel i9-10940X (3.30 GHz) on 14 CPUs. The average computational time of the falling mass simulation was 10 min; while the average computational time of the FSI simulation was 48 h.

### 2.2 Energetic analysis

All mechanical motion is the result of some form of energy transformation. Work is a measure of the energy transfer that occurs when an object is moved over a distance by an external force. The sign of the work done on an object determines if energy is transferred in or out of the object. A force applied to an object in the opposite direction to its motion will tend to slow it down, and thus it would drew kinetic energy off the object. With energy leaving the object, the work done should be negative and *vice versa*.

In this study, we conducted an analysis of the mechanical work exchange occurring on the anterior corneal surface, as illustrated in Figure 1D, under the loading conditions detailed in Section 2.1. As explained earlier in the introduction, Corvis ST records by means of a Scheimpflug camera 140 images of the central section of the cornea over the 30 ms of the air jet. While the anterior corneal surface is visible at all times, the posterior corneal surface may experience distortion during corneal deformation, and cannot be acurately identified. Results of previous studies indicated statistically significant differences among the thickness values obtained from Pentacam and Corvis ST (Rajabi et al., 2022). Consequently, to make our energetic analysis more reliable, we focused on the anterior corneal surface rather than the posterior surface.

The work of external forces is computed in the y-direction due to the axi-symmetric nature of the eye model, rendering the total work along the x and z-axes zero.

In both the simulations, the cornea is loaded internally by the IOP. The work of the IOP in direction y on the anterior corneal surface can be calculated with Eq. (3).

$$\begin{array}{c} {Work}_{\text{IOP}}=\overset{\text{increments}}{\underset{\text{j}=1}{\sum }}{Work}_{\text{IOP}}^{\text{j}}\\ {Work}_{\text{IOP}}^{\text{j}}=\overset{\text{nodes}}{\underset{\text{i}=1}{\sum }}\frac{\left(F_{{IOP}_{\text{i}}}^{\text{j}}+F_{{IOP}_{\text{i}}}^{\text{j}-1}\right)\cdot \left(u_{\text{i}}^{\text{j}}-u_{\text{i}}^{\text{j}-1}\right)}{2}\\ F_{{IOP}_{\text{i}}}^{\text{j}}=IOP\left(\Delta t^{\text{j}}\right)\cdot {area}_{\text{i}}\left(\Delta t^{\text{j}}\right) \end{array}$$ (3)

where at increment j of the simulation: ${area}_{\text{i}}\left(\Delta t^{\text{j}}\right)$ is the area associated to the node i and $u_{\text{i}}^{\text{j}}$ is its displacement in direction y. Since the IOP exerted by the humors is modelled as homogenous in space, it has the same interpolated value over the nodes.

On the external side of the anterior surface, the region of interest is loaded by different conditions. In case of the mass falling under the effect of gravity, the external work transmitted to the cornea corresponds to the total mechanical energy of the mass, which is the sum of its kinetic and potential energies.

In case of the air puff, the external work due to the air is given by:

$$\begin{array}{c} {Work}_{\text{AIR}}=\overset{\text{increments}}{\underset{\text{j}=1}{\sum }}{Work}_{\text{AIR}}^{\text{j}}\\ {Work}_{\text{AIR}}^{\text{j}}=\overset{\text{nodes}}{\underset{\text{i}=1}{\sum }}\frac{\left(F_{{\text{AIR}}_{\text{i}}}^{\text{j}}+F_{{AIR}_{\text{i}}}^{\text{j}-1}\right)\cdot \left(u_{\text{i}}^{\text{j}}-u_{\text{i}}^{\text{j}-1}\right)}{2}\\ F_{{AIR}_{\text{i}}}^{\text{j}}=P_{{AIR}_{\text{i}}}\left(\Delta t^{\text{j}}\right)\cdot {area}_{\text{i}}\left(\Delta t^{\text{j}}\right) \end{array}$$ (4)

where at increment j of the simulation: $P_{{AIR}_{\text{i}}}\left(\Delta t^{\text{j}}\right)$ is the equivalent air pressure on the node i on the corneal anterior surface obtained by the software; ${area}_{\text{i}}\left(\Delta t^{\text{j}}\right)$ is the area associated to that node and $u_{\text{i}}^{\text{j}}$ is its displacement in direction y. The air pressure is calculated by the software on the center of the cell and then projected to the nodes of the mesh. The air pressure over the corneal anterior surface is not constant neither homogeneous; it depends on the corneal deformation as depicted in Figure 1D.

## 3 Results

### 3.1 Energetic analysis of the deformation of the eye under the effect of a falling mass

In this section, the results of the energy analysis of the mass falling onto the eye are presented.

**FIGURE 2:**
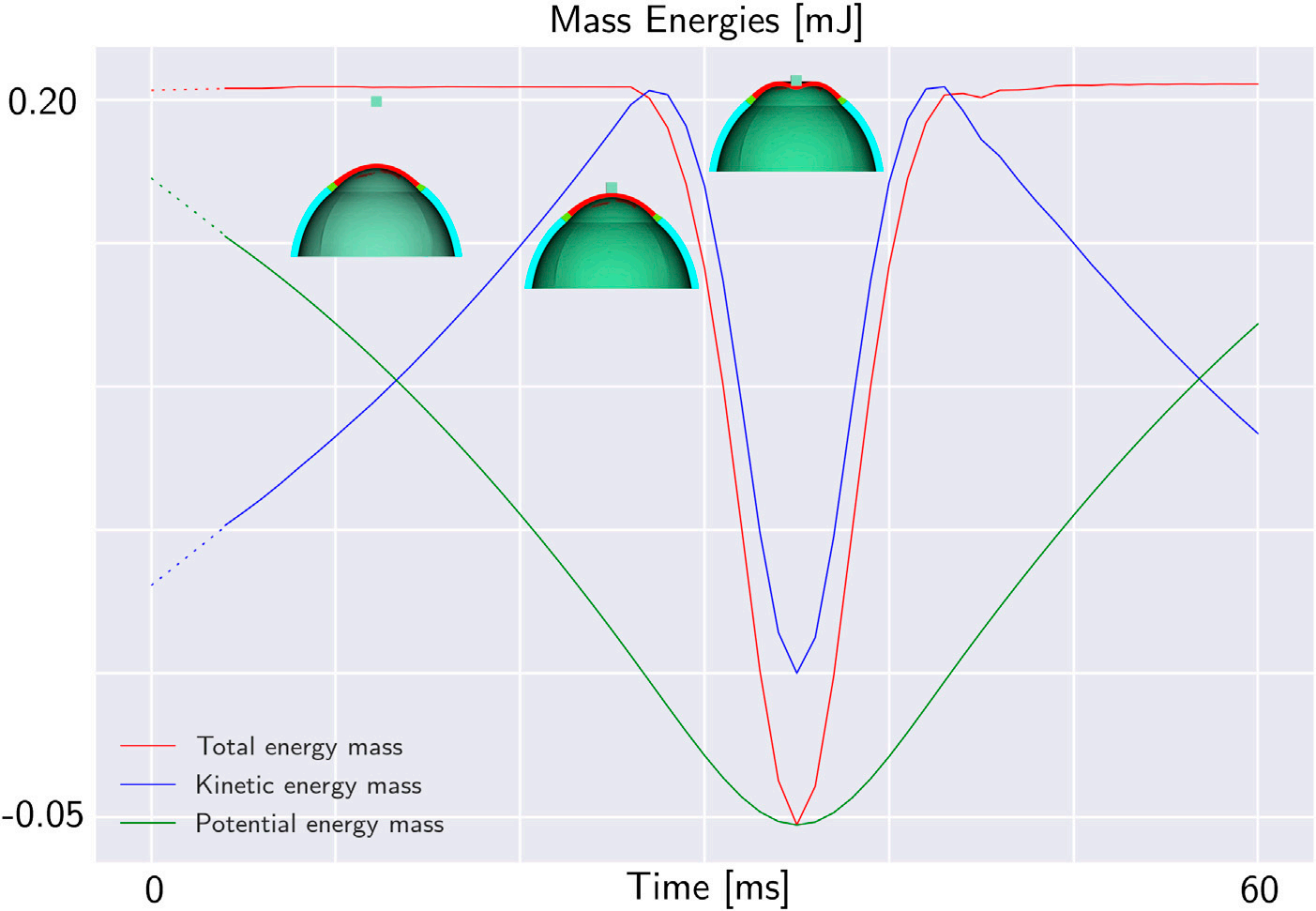
Total mechanical energy of a mass falling under the effect of gravity on the eye. The potential energy is in green, the kinetic energy in blue and the total mechanical energy in red. In the first 25 ms, the mass is falling without impacting the eyeball. Until the impact, the total mechanical energy of the mass is constant. When the mass impacts the eye, its mechanical energy decreases. At 35 ms, the maximum deformation of the eye occurs, then the structure returns to its original configuration.

Initially, the mass is at a certain height with respect to the eye and starts falling under the force of gravity. As it falls, its kinetic energy increases due to its increasing speed while its potential energy decreases. In the initial part of the plot in Figure 2, the curves are depicted with dashed lines, because numerically it is not possible to impose to the mass a sudden acceleration equal to the gravitational acceleration. Numerically, the mass begins with zero acceleration, and in the initial instants, it dynamically reaches the gravitational acceleration. For this reason, when the gravitational acceleration is reached (at 4 ms), the velocity (and therefore the kinetic energy of the mass) is not zero. When the system stabilizes, the sum of the potential and kinetic energies is constant as depicted with the red line in Figure 2.

**FIGURE 3:**
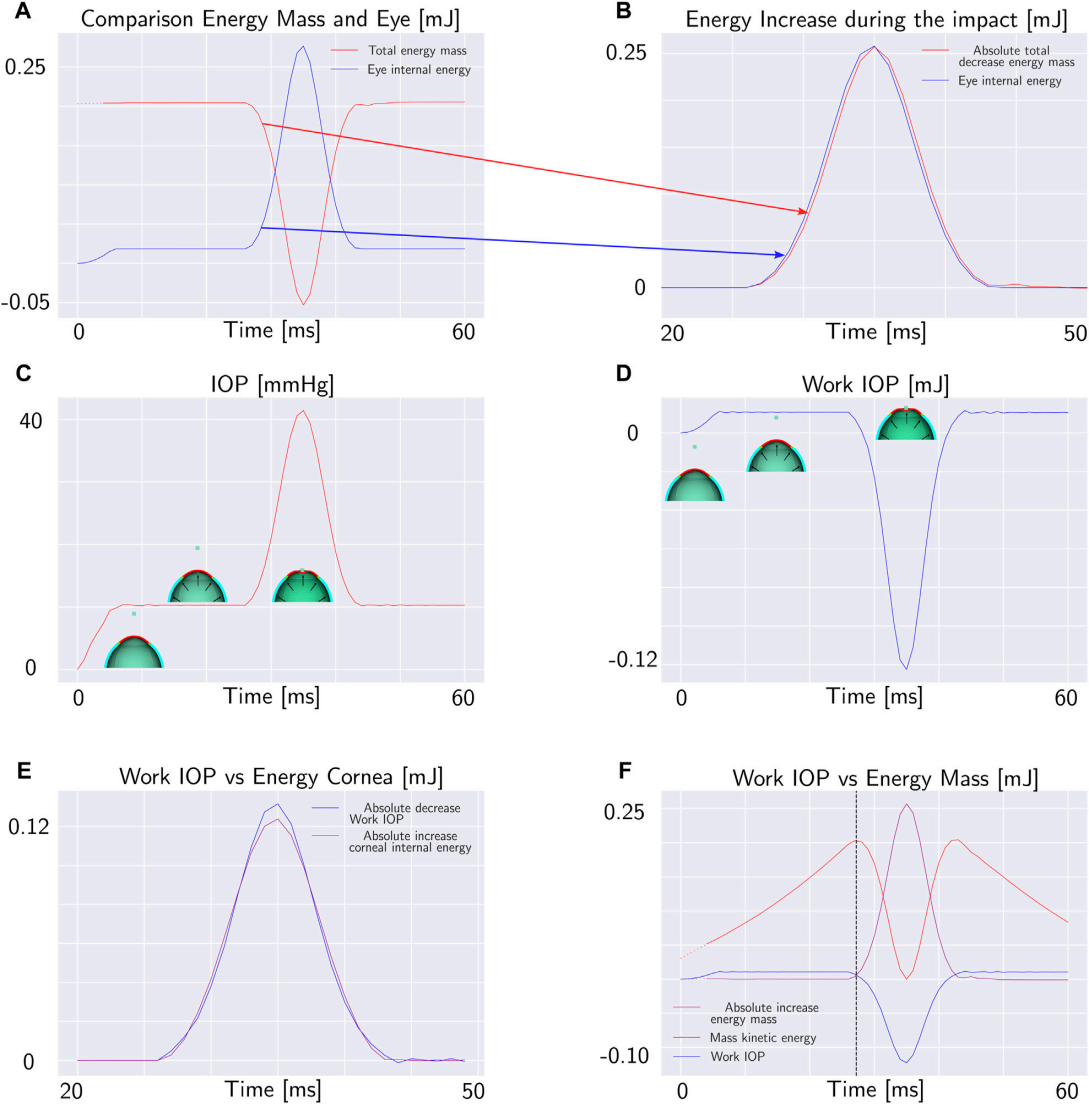
Mass falling under the effect of gravity on the eye: **(A)** Total energy of the mass and total internal energy of the eye. **(B)** Increase of the internal energy of the eye and absolute value of the decrease of the total energy of the mass during the impact. **(C)** Pressure of the fluid cavity during the impact. **(D)** Work of the internal fluid on the anterior corneal surface. **(E)** Internal energy of the cornea and work of the internal fluid (with opposite sign). **(F)** Work of the internal fluid, total energy of the mass and kinetic energy of the mass. In the intersection point between the total energy of the mass and work of the internal fluid, the kinetic energy is maximum.

When the mass contacts the external surface of the eye-globe, it starts to deform the object. For this reason, the total energy of the mass decreases. The total mechanical energy of the mass along with its potential and kinetic energy during the impact are depicted in Figure 2. At the point of impact, the energy of the mass is transmitted to the eye where it is stored in the form of elastic energy. Figure 3A. Plots both the total energy of the mass and the total internal energy of the eye. The total internal energy of the eye increases in the first 5 ms corresponding to the eye pressurization. Then, it remains constant before experiencing an important increase during the impact. Since there is no energy dissipation, the absolute decrease of the energy of the mass is equal to the increase of the internal energy of the eye as shown in Figure 3B.

Meanwhile, as the mass falls onto eye-ball, the pressure inside the chamber of the eye increases uniformly in all directions, following the principles of hydrostatic pressure (Figure 3C). This pressure acts on the surrounding walls of the container, causing it to expand to accommodate the increased pressure. The pressure in the fluid exerts a mechanical work on the anterior surface of the cornea calculated with Eq. 3. The work of the IOP increases as the pressure rises during the pressurization phase. In the pressurization phase, the work is positive because it is done by the fluid expanding the surrounding walls. After the impact with the mass, the work done by the fluid starts to decrease (Figure 3D). This decrease occurs because the displacement of the cornea is in the opposite direction to the IOP force.

On the other hand, during the impact of the mass with the eye, the internal energy of the cornea increases. Since the cornea is much more compliant than the sclera, the variation in the internal energy of the cornea during the impact is very close to the work exerted by the IOP as shown in Figure 3E. In the first phase of the impact, all the corneal deformation is converted into an increase of IOP. Moreover, even if the variation in the elastic energy of the cornea during the impact generally is an unknown fraction of the variation of the elastic energy of the eye-ball, depending on the compliance of the sclera and on the diameter of the cornea relative to the diameter of the sclera, in the first phase of the simulation this fraction is close to 1. For this reason, since the corneal deformation energy fully transmits energy to the internal fluid, we can analyze the energy balance only by considering the work done by the IOP and the external energy.

When the work of the IOP becomes equal to the total mechanical energy acting on the mass, the net force on the mass becomes zero and the mass stops accelerating. We will concentrate our analysis on the time at which the works acting on the corneal anterior surface are equal. The intersection point between the work of the IOP and the decrease in the total energy of the mass is the point of maximum kinetic energy and, therefore the point of maximum velocity of the mass as depicted in Figure 3F. The maximum velocity of the mass is equal to the maximum velocity of the corneal apex because the mass falls over the corneal apex; therefore when the works acting on the anterior corneal surface are equal, the apex velocity is maximum.

Remark 1 *The mechanical energy of the mass (kinetic and potential) is converted in deformation of the eyeball, in particular, in the first phase of the deformation it is converted in deformation energy of the cornea.*

Remark 2 *The deformation of the cornea during the impact is converted in increase of IOP.*

Remark 3 *The time when the works acting on the anterior corneal surface are equal occurs when the velocity of the apex is maximum.*

### 3.2 Energetic analysis of the deformation of the eye under the effect of an air jet of corvis ST

According to the results obtained by the mass falling model, we propose to study the cornea subjected to the air puff of Corvis ST at the time when the works acting on the anterior corneal surface are equal. Based on those previous results, all the external energy is converted in increasing the IOP, therefore during the first part of the deformation, the energy transfer only depends on the IOP. In this section, the balance between the work of the air puff and the work of the IOP on the corneal anterior surface is calculated, to verify that the air puff effect on the eye is the same as the mass. To achieve this aim, the outline of the FSI simulation described in Section 2.1 is used with multiple combinations of mechanical properties of corneal tissue, IOP and thickness as presented in Table 1. Actually, the displacement field, strain and stress states of the FSI depend on the IOP, the mechanical properties of the corneal tissue and corneal geometry as demonstrated in our previous work (Redaelli et al., 2022) because it is a strongly coupled problem. In the simple problem with the mass, the energy of the mass does not depend on the eye deformation, while in this case, the air pressure over the corneal apex depends on the corneal deformation as presented in Figure 1D. We performed the same FSI simulation sixteen times, with each repetition involving a variation in one parameter in order to reproduce the NCT test for different patients. In the first thirteen simulations, the corneal geometry was the same with a Central Corneal Thickness (CCT) of 558 μm, the varying parameters of these simulations were the parameters describing the mechanical properties of the corneal tissue and the IOP. In the last three simulations, also the corneal geometry changed, with different corneal thickness. The parameters used in the simulations are listed in Table 1. For each simulation, the air pressure over the corneal anterior surface, the corneal displacement and the IOP were measured as output during the air puff. These quantities were used to calculate the work of the IOP and the work of the air pressure over the corneal anterior surface with Eqs 3, 4.

**TABLE 1:** Parameters of the mechanical model of the corneal tissue, intraocular pressure (IOP) and central corneal thickness (CCT) for each simulation. $C_{10}$ represents the collagen matrix stiffness in MPa , $k_{1}$ the fibers stiffness in MPa and $k_{2}$ the fibers non linearity [−].

Simulation	$C_{10}$ [MPa]	$k_{1}$ [MPa]	$k_{2}$ [-]	IOP [mmHg]	CCT [μm]
1	0.045	0.027	180	10	558
2	0.045	0.027	180	15	558
3	0.045	0.027	180	20	558
4	0.045	0.027	180	25	558
5	0.045	0.027	180	30	558
6	0.0675	0.027	180	15	558
7	0.0225	0.027	180	15	558
8	0.045	0.0405	180	15	558
9	0.045	0.0135	180	15	558
10	0.045	0.027	270	15	558
11	0.045	0.027	90	15	558
12	0.035	0.13	1,000	15	558
13	0.01	0.015	100	15	558
14	0.045	0.027	180	15	484
15	0.045	0.027	180	15	525
16	0.045	0.027	180	15	600

#### 3.2.1 Pressurization phase

**FIGURE 4:**
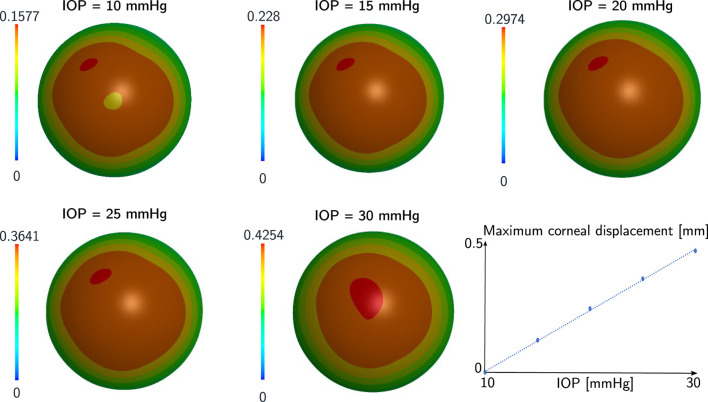
Displacement in mm of the anterior corneal surface at the end of pressurization for the first five cases of the sensitivity analysis. The mechanical properties and the geometry of the cornea are the same in each case, the IOP varies. The corneal displacement at the end of pressurization had a linear relationship with the IOP.

Initially, we analyzed the first five simulations characterized by the same CCT and mechanical properties and different IOP (ranging between 10 and 30 mmHg). The zero-pressure configuration of the eye varies in each simulation since we started from the same pressurized configuration. For this reason, the displacement of the anterior surface of the cornea from the zero pressure configuration to the end of pressurization is different in each case. The displacement contour at the end of the pressurization is depicted in Figure 4.

**FIGURE 5:**
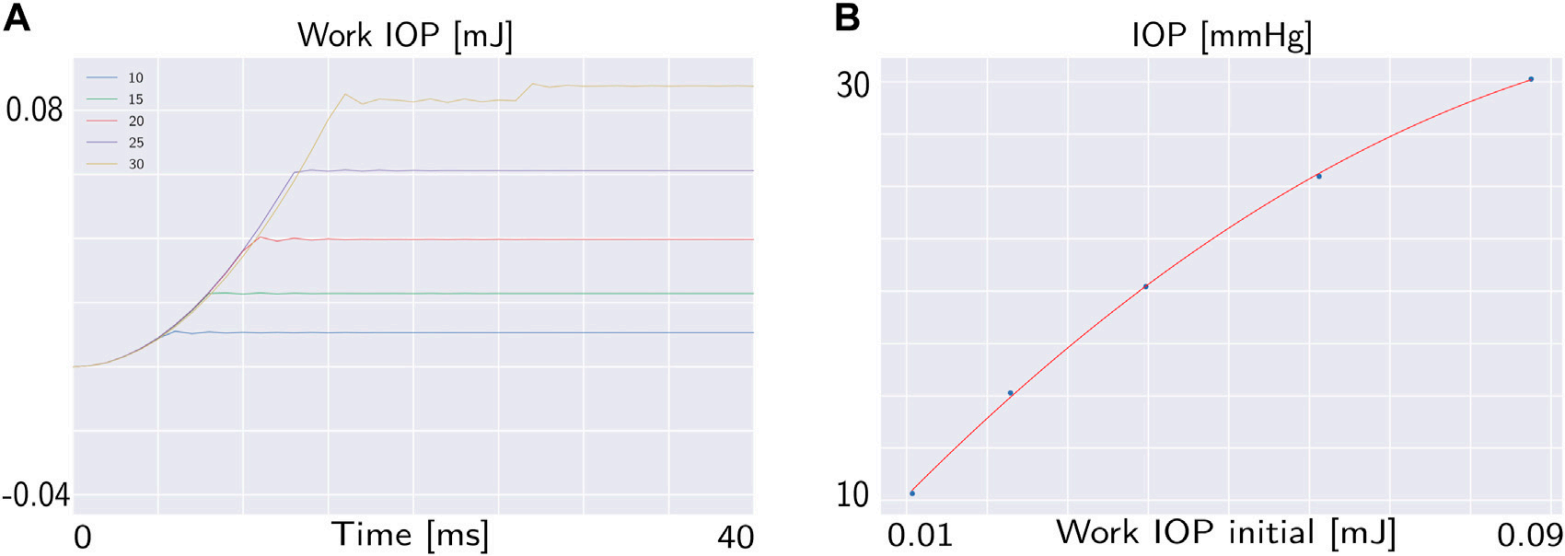
**(A)** Work of the IOP during the pressurization phase for different IOP. **(B)** Relationship between the work of the IOP at the end of pressurization and the IOP.

The deformation state of the cornea at the end of pressurization is different in each case since the displacement at the end of pressurization depends on the IOP. In particular, the maximum displacement in each case has a linear relationship with the IOP as depicted in Figure 4. The work of the IOP at the end of pressurization depends on the IOP of the patient and on the displacement of the anterior surface of the cornea. For this reason, the IOP has a quadratic relationship with the work of the IOP at the end of pressurization as depicted in Figures 5A,B.

In particular, under the mechanical properties of the corneal tissue analysed, the relationship between the IOP and the work of the IOP at the end of pressurization is defined by Eq. 5:

$$\begin{array}{l} \text{IOP}=\left(-1.69\cdot 10^{3}\right)\cdot {\text{Initial work IOP}}^{2}+\left(4.21\cdot 10^{2}\right)\\ \cdot \text{Initial work IOP}+6.18\\ R^{2}=0.98 \end{array}$$ (5)

#### 3.2.2 Air jet phase

**FIGURE 6:**
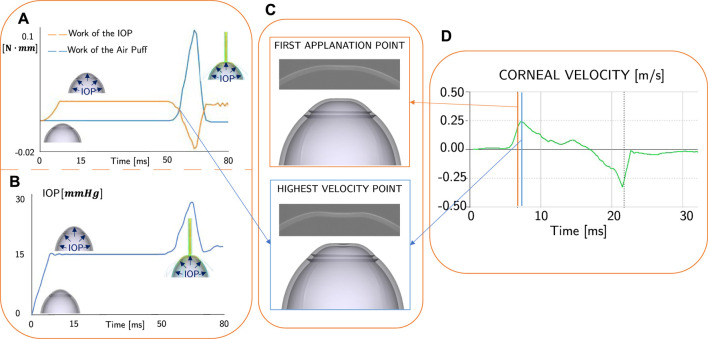
**(A)** Work of the air puff (in blue) and work of the IOP (in orange) calculated from the FSI simulation: the intersection point of the curves is the interest of this analysis. **(B)** Evolution of the IOP during the simulation: when the air puff impact the eye, the IOP increases. **(C)** Clinical and numerical comparison between the first applanation point and the highest velocity point. **(D)** Clinical result: apex velocity as output of Corvis ST: the first applanation time (orange line) does not coincide with the highest velocity time (blue line).

In Figure 6 the evolution of the work of the air puff and the work of the IOP during the impact are represented for one of the sixteen cases from the sensitivity analysis (Table 1).

As illustrated in Figure 6A, the IOP’s work increases in the first 10 ms as the eye undergoes pressurization from its initial zero-pressure state. In the subsequent stabilization step, both the work of the air puff and the work of the IOP remain constant. At 50 ms, the corneal surface is impacted by the air puff, resulting in deformation in the direction opposite to that of the IOP but in the same direction as the air puff pressure. Additionally, the IOP increases during the air puff, as depicted in Figure 6B and reported in our previous work (Redaelli et al., 2022) because the fluid is incompressible. Conversely, the work of the air puff increases during the impact (Figure 6A), while the work of the IOP decreases. There are two intersection points of the curves, which correspond to the time of maximum velocity of the apex. This time is different from the time of the first applanation, as shown in Figures 6C,D. The time difference is not always the same since it depends on the corneal structural properties, the range of difference is between 1 and 5 ms.

**FIGURE 7:**
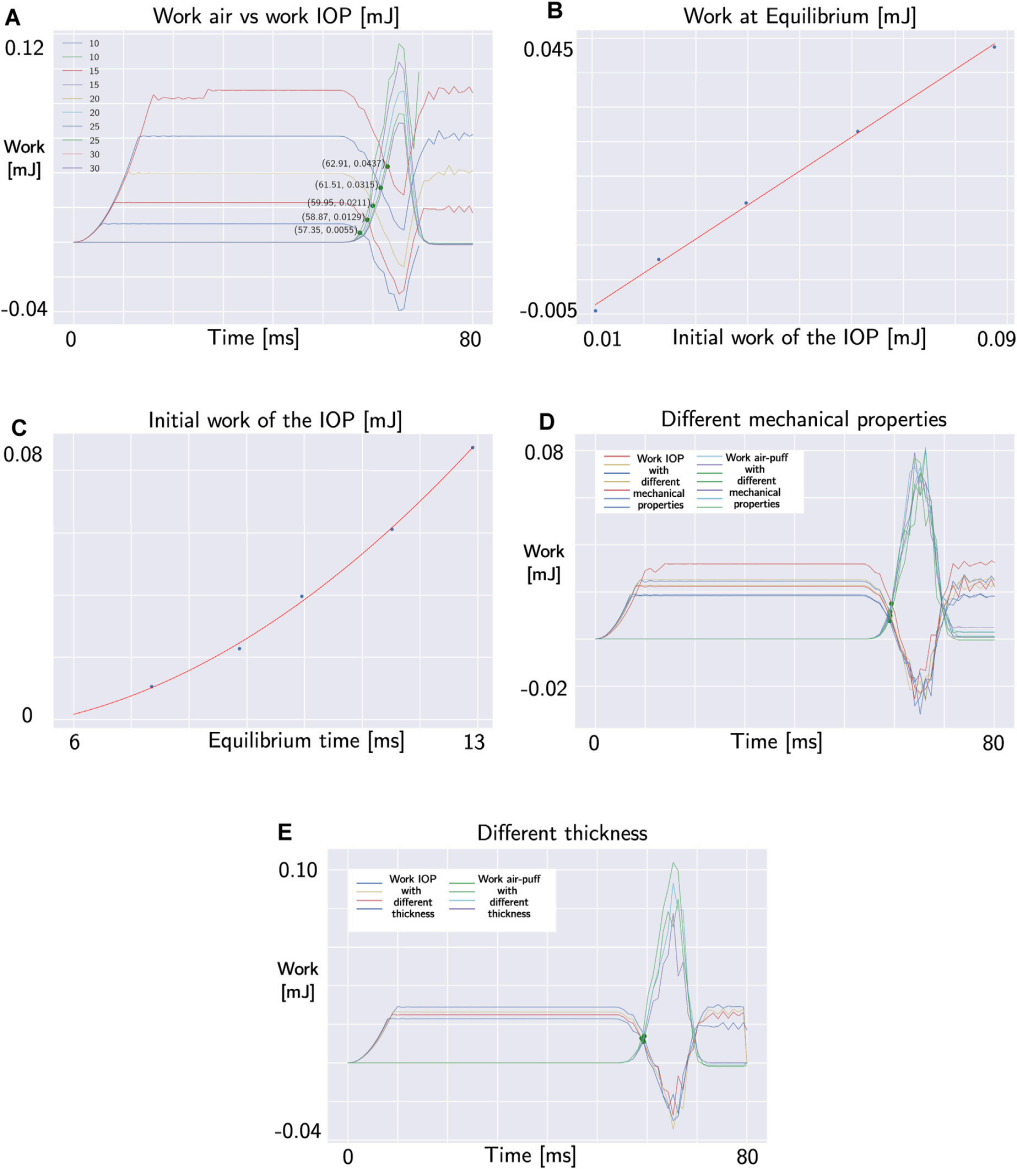
**(A)** Intersection points between internal and external work in the simulations with different IOP. **(B)** Linear relationship between the initial work of the IOP and the work at intersection (both in mJ). **(C)** Quadratic relationship between the time of maximum velocity (in ms) and the initial work of the IOP (in mJ). **(D)** Intersection points between internal and external work in the simulations with different mechanical properties of the corneal tissue. **(E)** Intersection points between internal and external work in the simulations with different thickness.

Analyzing the first five simulations characterized by the same central corneal thickness (CCT) and mechanical properties but different IOP, we observe that the work of the IOP and the work of the air puff during the impact differ in each simulation, as shown in Figure 7A. Figure 7B demonstrates that, for the considered mechanical properties and thickness, the relationship between the initial work of the IOP and the work at the intersection is linear. Specifically, the work at the intersection is approximately half of the initial work. In particular, a quadratic relationship exists between the time of maximum velocity and the initial work of the IOP, as shown in Figure 7C and reported in Eq. 6.

$$\begin{array}{l} \text{Initial work IOP}\;\left[\text{mJ}\right]=\left(1.09\cdot 10^{-3}\right)\cdot {time}_{\text{max}-\text{vel}}^{2}+\left(-8.28\cdot 10^{-3}\right)\\ \cdot {time}_{\text{max}-\text{vel}}+1.19\cdot 10^{-2}\\ R^{2}=0.98 \end{array}$$ (6)

**FIGURE 8:**
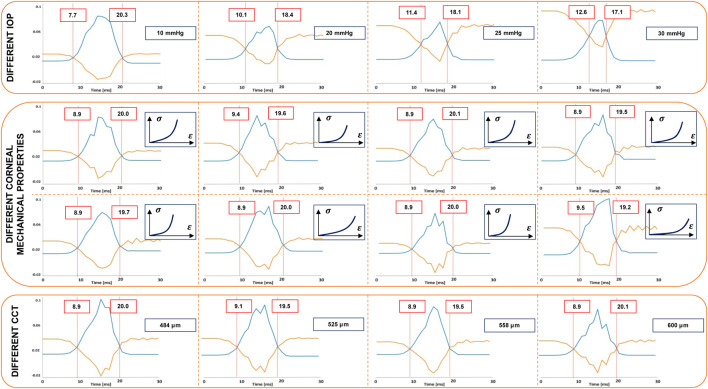
Time of intersection point between the work of the air puff (in blue) and the work of the IOP (in orange) in different FSI simulations (Table 1). This point of the cornea changes when the IOP changes. However, it does not depend on the mechanical properties of the corneal tissue and the central corneal thickness.

But what happens when we change the mechanical properties of the corneal tissue and the thickness of the cornea? (simulations 6–16 in Table 1). Changing both the mechanical properties of the corneal tissue and the thickness of the eye, the time of intersection between the work of the air puff and the work of the IOP does not change as demonstrated by the plots in Figures 7D,E. Therefore, this point does not depend on the geometry and mechanical proprierties of the cornea. The work of the air puff and the work of the IOP during the air puff for each simulation are reported in Figure 8.

Remark 4 *There exist a relationship between the maximum velocity time and the initial IOP.*

Remark 5 T*he mechanical properties of the corneal tissue and the corneal thickness does not influence the energetic balance at the beginning of the deformation.*

## 4 IOP estimation with corvis ST

### 4.1 Clinical analysis of the first applanation time and highest velocity time

**FIGURE 9:**
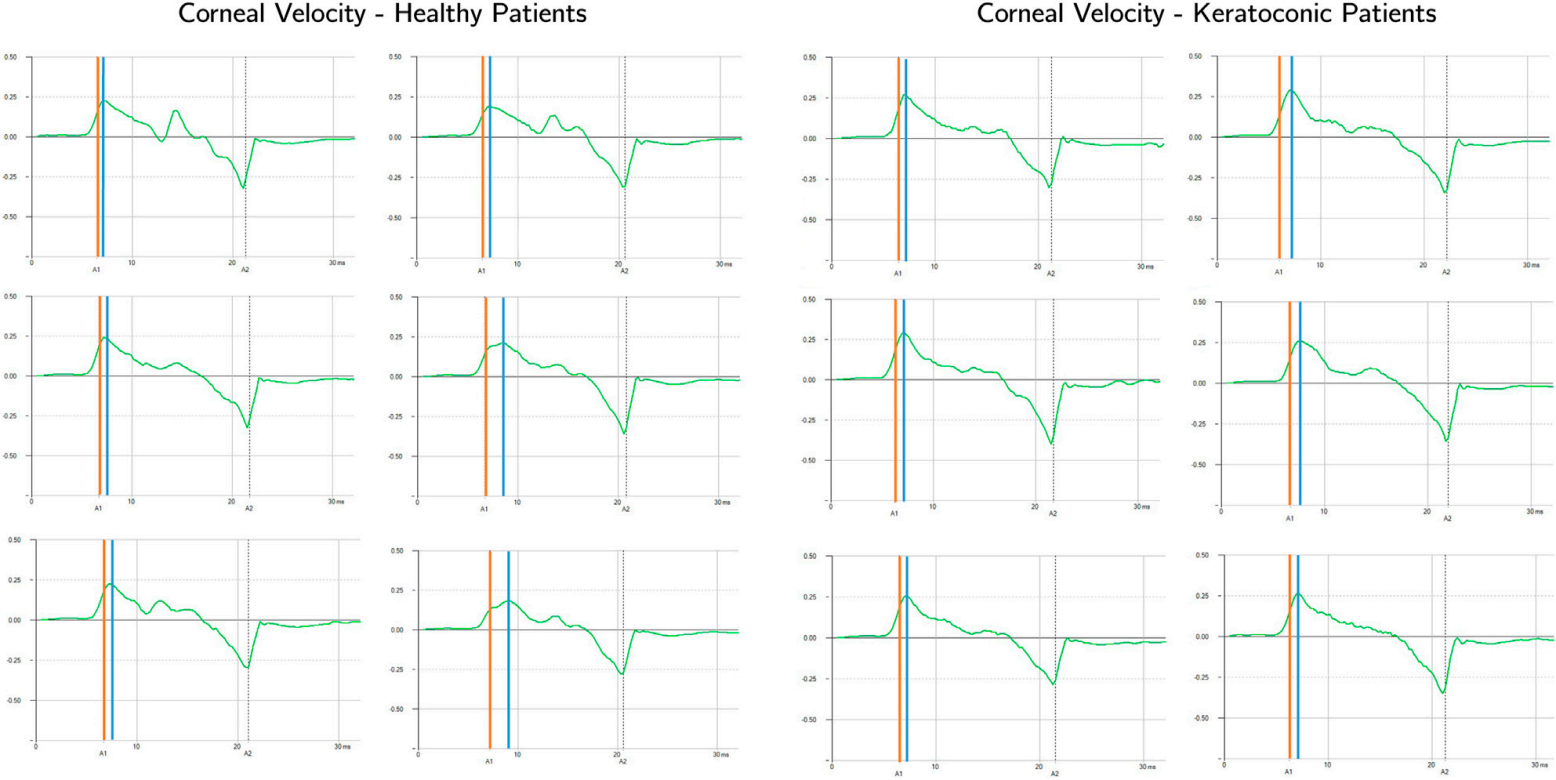
Apex velocity in time for six healthy and six keratoconic patients during Corvis ST. The first applanation time is highlighted with the orange vertical line while the highest velocity time is highlighted with the blue vertical line.

We presented in the previous sections, the results of numerical simulations of the Corvis ST test, to analyse the energy balance of the system. The outcomes of these simulations can be extrapolated to the clinical scenario, as the equipment captures the temporal evolution of apex velocity for each patient. In this section, we will delve into a detailed analysis of the apex velocity. The Corvis ST results of six healthy and six keratoconic corneas are presented in this section as an example. These data were used to evaluate if the first applanation time of the cornea and the point of maximum velocity are coincident. The data used were randomly selected from a previous study conducted at Antwerp University Hospital. The study was conducted according to the tenets of the Declaration of Helsinki and participants gave signed informed consent prior to measurement (reference number of the Antwerp University Hospital Ethical Committee: 17/12/136). The analysis of the apex velocity during Corvis ST examination from both healthy and keratoconic patients, revealed that the first applanation point, typically used as reference to measure the IOP *in-vivo*, does not correspond to the instant of highest velocity of the corneal apex as shown in Figure 9. Additionally, the time difference between the maximum velocity instant and the first applanation time varies among patients. After the first applanation point, the corneal velocity continues to increase, revealing that the cornea is subjected to a positive acceleration and force. The corneal velocity reaches its maximum value when the corneal acceleration is zero, therefore the force acting on the cornea is zero. After that, the corneal velocity decreases, reaching zero velocity at the highest concavity.

### 4.2 Methodology proposal

Given the results of Section 3, it is possible to propose a new procedure to estimate the IOP of a patient based on the time of maximum velocity of the corneal apex provided by Corvis ST. The new IOP will be called *wIOP* because it is based on the analysis of the works acting on the corneal anterior surface. Since the time at which the works acting on the corneal surface are equal does not depend on the mechanical properties of the corneal tissue and on the corneal thickness, this time can be used to estimate the wIOP. From the output data of Corvis ST, the velocity of the apex is known. The time of maximum velocity of the apex corresponds to the intersection time of the works and it is used to calculate the wIOP by means of Eq. 7 which is given by the combination of Eqs 5, 6.

$$\begin{array}{c} \text{wIOP}\;\left[\text{mmHg}\right]=\left(-2.01\cdot 10^{-3}\right)\cdot {{time}_{\text{max}-\text{vel}}}^{4}+\left(3.05\cdot 10^{-2}\right)\cdot {{time}_{\text{max}-\text{vel}}}^{3}\\ +\left(2.99\cdot 10^{-1}\right)\cdot {{time}_{\text{max}-\text{vel}}}^{2}-3.15\cdot {time}_{\text{max}-\text{vel}}+10.95 \end{array}$$ (7)

## 5 Discussion

**FIGURE 10:**
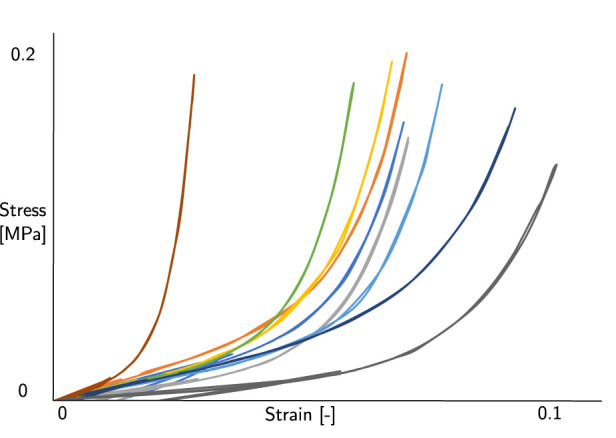
Stress strain curves of the corneal material tested in our simulations.

The point in which the external and internal forces are equal in NCT of Corvis ST was previously believed to be equal to first applanation point of the cornea (Sharma et al., 2020; Brusini et al., 2021; Silva and Lira, 2022). However, a closer inspection of the clinical results revealed that the first applanation point does not coincide with the point of highest velocity. The cornea’s deformation depends on the coupled effect of the IOP, the corneal thickness, the mechanical properties of the corneal tissue and the air pressure on the cornea (Ariza-Gracia et al., 2015; Redaelli et al., 2022). Consequently, the equilibrium point may not necessarily coincide with an applanated cornea. However, in the instant of highest velocity, the acceleration of the cornea is zero, indicating that the total force acting on the cornea is also zero. The FSI simulation presented in our previous work (Redaelli et al., 2022) predicts the air pressure on the corneal surface during the puff; in contrast with the output of the Corvis ST, which only provides the air pressure at the outlet of the device nozzle’s (Simonini and Pandolfi, 2016). Previous numerical works attempted to estimate the IOP removing the influence of corneal structural parameters. *Joda et al.* (Joda et al., 2016) and more recently *Eliasy et al.* (Eliasy et al., 2022) proposed an algorithm to correct the IOP estimated by Corvis ST trying to exclude the influence of CCT and age. However, their algorithm is based on structural numerical simulations in which the air pressure on the cornea does not vary depending on the mechanical properties and thickness of the patient. *Simonini et al.* (Simonini and Pandolfi, 2016) studied the influence of a varying air jet pressure on the output of the NCT, revealing that different air pressures on the cornea can lead to changes in the estimation of IOP. That work considered different pressure values on the cornea as a defined function of a structural simulation, independent on the corneal deformation. The Corvis ST tonometer nozzle consists of a tube with an inner diameter of 1–2 mm, and a distance of 11 mm between the nozzle and the eye is required, which affects the cross-sectional profile of the pressure applied to the eye. Due to the nozzle to eye distance and subsequent change in the pressure profile, the spatial and temporal profile of the airflow pressure on the cornea differs significantly from that of the internal device pressure (Oehring et al., 2021). *Oehring at al* (Oehring et al., 2021). investigated the spatial distribution of the Corvis ST airflow pressure using mapping grids. However, the pressure on the corneal surface was supposed to be only dependent on the distance, not on the corneal deformation. The novelty of our FSI is the evaluation of the air pressure on the corneal surface in each case with the possibility to calculate the work of the air puff in different scenarios. To the best of our knowledge, only *Zhang et al.* (Zhang et al., 2021) have investigated the NCT by means of an energetic approach but they considered a constant pressure on the corneal surface. The work of the air puff in *Zhang et al.* (Zhang et al., 2021) is assumed to be equal to the corneal internal energy. Our energetic analysis considers the energy balance of the anterior corneal surface at the intersection point between the work of the air puff and the work of the IOP. Both works are calculated on the anterior corneal surface, so the thickness of the eye is not considered and does not influence the results, as supported by the analysis of different CCT shown in Section 3.2. We conducted an analysis of the energy balance in the *y* direction, since the thickness of the structure is negligible with respect to its surface, the mechanical properties of the cornea do not play a role in the results. Our analysis of various mechanical properties in Section 3.2 supports this non-dependency. Figure 10 depicts the stress-strain curves of the corneal materials we tested which are consistent with other previous experimental studies (Elsheikh et al., 2008; Xiang et al., 2018; Liu et al., 2020). *Xue et al.* (Xue et al., 2018) found that under physiological IOP, the corneal stress is about 0.02 MPa, which is consistent with the stress strain curves we tested in our simulations.

We are aware that our research has some limitations. First of all, the FSI simulations conducted and the clinical evaluation was conducted for patients with a regular geometry. Further studies should be conducted with the aim to study the air pressure distribution on the corneal surface in case of non-symmetric corneal geometries, for example, in keratoconic patients, where the bulging of a region of the cornea has the consequence of an non symmetric geometry. Or other studies should be conducted in analyzig the nergy balance in case the air jet does not impact exactly the apex of the cornea. Furthermore, an *in vitro* validation of the procedure should be conducted. The problem with *in vitro* validation is that we should have a very sensitive sensor (with a sampling similar to the one of the camera) capable of measuring the IOP changes in the experimental set up during the air puff. To the best of author knowledge, some experimental set-up with Corvis ST have been proposed to estimate the influence of the IOP on corneal deformation during the air puff (Kling and Marcos, 2013; Bekesi et al., 2016a; Bekesi et al., 2016b; Bekesi et al., 2017; Eliasy et al., 2018). However, the pressure transducers used in these experiments does not capture the IOP change which occurs during the air puff.

In conclusion, the significance of our novel pressure estimation lies in two key aspects. Firstly, the method provides a more realistic and real-time assessment of a patient’s intraocular pressure (IOP) based on the results of Corvis ST, decoupling it from the mechanical properties of the cornea. This real-time capability enhances the dynamic monitoring of IOP, allowing for prompt and responsive adjustments in clinical interventions.

Secondly, once an accurate estimate of IOP is obtained, it can serve as a foundational parameter for further investigations. Specifically, this IOP data can be leveraged in conjunction with other methodologies to estimate the mechanical properties of the cornea. This dual-pronged approach addresses an emerging challenge in ophthalmology, offering a comprehensive understanding of both IOP and corneal biomechanics. By advancing our ability to assess these interconnected factors in real-time, our methodology contributes to a more nuanced and comprehensive approach to ocular health diagnostics.

## 6 Conclusion

The corneal deformation induced by the air puff is influenced by IOP, CCT, mechanical properties of the corneal tissue and the force exerted by the air puff. Our proposed methodology in this study focuses on isolating and accurately estimating the IOP, excluding the effects of the aforementioned factors. By decoupling the contribution of these variables, our approach provides a more reliable assessment of IOP. The new wIOP can be useful in advancing our understanding of ocular biomechanics and establishing a more accurate foundation for diagnostic and therapeutic interventions.

## Data Availability

The original contributions presented in the study are included in the article/Supplementary Material, further inquiries can be directed to the corresponding author.
